# Extended Multilocus Sequence Analysis to Describe the Global Population Structure of the Genus *Brucella*: Phylogeography and Relationship to Biovars

**DOI:** 10.3389/fmicb.2016.02049

**Published:** 2016-12-21

**Authors:** Adrian M. Whatmore, Mark S. Koylass, Jakub Muchowski, James Edwards-Smallbone, Krishna K. Gopaul, Lorraine L. Perrett

**Affiliations:** FAO/WHO Collaborating Centre for Reference and Research in Brucellosis and OIE Brucellosis Reference Laboratory, Department of Bacteriology, Animal and Plant Health AgencyAddlestone, UK

**Keywords:** *Brucella*, brucellosis, multilocus sequence, molecular typing, zoonosis

## Abstract

An extended multilocus sequence analysis (MLSA) scheme applicable to the *Brucella*, an expanding genus that includes zoonotic pathogens that severely impact animal and human health across large parts of the globe, was developed. The scheme, which extends a previously described nine locus scheme by examining sequences at 21 independent genetic loci in order to increase discriminatory power, was applied to a globally and temporally diverse collection of over 500 isolates representing all 12 known *Brucella* species providing an expanded and detailed understanding of the population genetic structure of the group. Over 100 sequence types (STs) were identified and analysis of data provided insights into both the global evolutionary history of the genus, suggesting that early emerging *Brucella abortus* lineages might be confined to Africa while some later lineages have spread worldwide, and further evidence of the existence of lineages with restricted host or geographical ranges. The relationship between biovar, long used as a crude epidemiological marker, and genotype was also examined and showed decreasing congruence in the order *Brucella suis* > *B. abortus* > *Brucella melitensis*. Both the previously described nine locus scheme and the extended 21 locus scheme have been made available at http://pubmlst.org/brucella/ to allow the community to interrogate existing data and compare with newly generated data.

## Introduction

Brucellosis remains one of the world's most important zoonotic diseases and continues to have a significant impact on animal and human health in much of the world particularly in South America, Southern Europe, Africa, the Middle East, and much of Asia (Pappas et al., 2006). Recent years have seen the beginning of an expansion of the genus *Brucella* (Whatmore, 2009) from the six classically identified species [*Brucella abortus* (cattle), *Brucella melitensis* (sheep and goats), *Brucella suis* (pigs, hares, reindeer), *Brucella canis* (dogs), *B. ovis* (sheep), and *B. neotomae* (rodents)] with the description of six additional species (*Brucella microti* (voles), *Brucella pinnipedialis* (pinnipeds), *Brucella ceti* (cetaceans), *Brucella papionis* (baboons), *Brucella vulpis* (foxes), and *Brucella inopinata* (isolated from a human case—natural host unknown). Although *Brucella* have long been known to represent a highly homogeneous genus, even for a time being classified as a monospecific genus (Verger et al., 1985), the known diversity of the group was extended greatly by the description of this latter species (De et al., 2008; Wattam et al., 2012) and *B. vulpis* (Hofer et al., 2012; Scholz et al., 2016). These groups are often described as “atypical” in the literature reflecting their genetic separation from classical species. A number of other isolates that await formal taxonomic description originating from sources as diverse as humans, frogs, fish, and additional rodents will likely extend diversity within both the classical group and newly emerging “atypical” *Brucella* in the near future (Tiller et al., 2010a,b; Godfroid et al., 2011; Eisenberg et al., 2012; Scholz and Vergnaud, 2013; Whatmore et al., 2015; Eisenberg et al., 2016).

Of the species described above *B. melitensis, B. abortus*, and *B. suis* are the most significant in terms of both animal and human disease impact. Where achieved, control has reflected a combination of measures including animal vaccination and/or control strategies (“test and slaughter”) along with improved food hygiene standards. International trade standards are applied as part of efforts to help control spread of these pathogens (World Organisation for Animal Health, 2011). *B. canis* and *B. ovis* are less economically significant pathogens of animals and, of these species, only *B. canis* is known to cause rare infections of humans (Marzetti et al., 2013). The significance of *Brucella* infection in the marine ecosystem remains uncertain (Nymo et al., 2011) and there is only evidence of one genotype causing serious, but rarely reported, naturally acquired infections of humans (Whatmore et al., 2008). *B. inopinata* has been confined to a single human case with no reservoir of infection identified and, while *B. microti* appears highly virulent in its natural host and other rodents (Jiménez de Bagüés et al., 2010), any pathogenic potential for man remains to be elucidated.

The three major species, *B. melitensis, B. abortus*, and *B. suis* are divided into biovars by a biotyping scheme that for many years has been the gold standard for *Brucella* characterisation at both the species and subspecies level. The scheme, based on a combination of growth characteristics, biochemical reactions, serotyping and bacteriophage typing, distinguishes the six classical species and further subdivides the major species into seven (*B. abortus*), five (*B. suis*) or three (*B. melitensis)* biovars, respectively. The homogeneity of the classical species meant that slow progress was made in identifying molecular markers to consistently define species and particularly to type at the subspecies level. Thus, in spite of the obviously limited resolution offered, biovars became commonly used epidemiological markers with particular biovars associated with certain geographical areas or certain hosts. However biotyping in its traditional form is expensive, time-consuming, involves hazardous culture and its rather subjective nature means it needs to be carried out by highly experienced scientists and is likely prone to inconsistency between laboratories. Further this traditional method is likely to become increasingly less relevant as the genus expands and new species emerge that diverge from classical criteria. As more molecular diversity has become apparent with technological advances biotyping is increasingly being replaced by the use of frontline molecular tools notably various diagnostic PCRs based on genomic deletions or SNPs (Foster et al., 2008; Gopaul et al., 2008, 2010; López-Goñi et al., 2008). However the performance of such tools is critically dependent on their design being based on an accurate and comprehensive understanding of the population genetic structure of the groups they are designed to separate (Keim et al., 2004).

This paper updates current understanding of the population structure of *Brucella* based on an extended (from an existing nine locus scheme—Whatmore et al., 2007) and extensive multilocus sequence analysis (MLSA) examining 21 independent genetic loci applied to a geographically and temporally diverse collection of over 500 *Brucella* isolates. The data significantly add to understanding of the genetic diversity of the group providing insights into evolutionary history, phylogeography, the relationship between genotype and biovar and provides a comprehensive framework to further understanding of these issues, for the future placement of newly emerging or atypical isolates and for the rational design of robust and accurate rapid diagnostic assays.

## Methods

### Bacteriology

Isolates were minimally cultured on serum dextrose agar and DNA preparations or crude lysates were prepared by standard procedures described previously (Whatmore et al., 2006) to serve as PCR template. Biotyping was undertaken by standard approaches as described elsewhere (Alton et al., 1988; Whatmore, 2009).

### MLSA

A 21 locus MLSA scheme (BruMLSA21) was developed by identifying an additional 12 informative housekeeping gene loci and adding these to the BruMLSA9 scheme described earlier (Whatmore et al., 2007). Loci and corresponding primers used in the scheme are described in Table S1. These were designed such as that identical PCR procedures could be applied to all 21 loci and thus PCR parameters and downstream purification and sequencing procedures were all as described previously (Whatmore et al., 2007). BruMLSA21 profiles of 508 isolates representing all 12 *Brucella* species (172 *B. abortus*, 84 *B. melitensis*, 100 *B. suis*, 24 *B. canis*, 11 *B. ovis*, 73 *B. ceti*, 20 *B. pinnipedialis*, 7 *B. microti*, 3 *B. neotomae*, 2 *B. papionis*, 2 *B. vulpis*, 1 *B. inopinata* and 9 unclassified *Brucella* isolates) were obtained and used in the analysis described here. Sequences of two strains *B. abortus* 9–941 and *B. abortus* 2308 were extracted from previously published whole genome sequences (Chain et al., 2005; Halling et al., 2005).

### Data analysis

A representative strain of each genotype (sequence type or ST) was used for phylogenetic analysis. Sequences of the 21 loci were concatenated to produce a 10,257 bp sequence (including indels) for each genotype. Phylogenetic analysis was performed with the MEGA software, Version 5.2 (Tamura et al., 2011). Neighbour joining trees were constructed using the Jukes-Cantor model and the percentage bootstrap confidence levels of internal branches were calculated from 1000 resamplings of the original data.

Minimum spanning trees were constructed in Bionumerics using the predefined template and the categorical coefficient. STs are represented by circles and the size of the circle is indicative of the number of isolates of that particular type. The coloring inside the circles indicates the *Brucella* species (**Figure 2**) or geographical origin (**Figures 4, 6, 8**). The different line types connecting genotypes reflect different numbers of shared loci as described in figure legends. The maximum neighbor difference used to create complexes indicated by the gray shading is given in individual figure legends.

### PubMLST

In common with MLST/MLSA schemes for most other bacteria, an open and expandable database has been established at the PubMLST website using the Bacterial Isolate Genome Sequence Database (BIGSdb) platform (Jolley and Maiden, 2010). Databases containing allele descriptions and allelic profiles for both BruMLSA9 (Whatmore et al., 2007) and the extended BruMLSA21 described here, and a corresponding isolate database, are available at http://pubmlst.org/brucella/ where data can be interrogated and submissions of new data are encouraged. All BruMLSA21 data that forms the basis of the analyses described in this communication, as well a significant volume of previously undescribed BruMLSA9 data, have been deposited in these databases.

### Other molecular testing

Confirmation of isolates as *B. abortus* vaccine strains S19 and RB51 was performed using previously described PCR assays (Sangari and Agüero, 1994; Vemulapalli et al., 1999) and confirmatory SNP based typing (Gopaul et al., 2010).

## Results and discussion

### Extension of existing MLSA scheme

A previously described MLSA scheme based on nine loci (BruMLSA9) provided limited resolution dividing 161 *Brucella* isolates into only 27 STs reflecting the relative genetic homogeneity of the classical *Brucella* species (Whatmore et al., 2007). In order to provide a supplementary scheme with increased resolution this scheme was extended by the addition of fragments representing 12 additional informative housekeeping genes to characterize 21 distinct loci in total. Here we describe its application to a temporally and geographically diverse collection of over 500 *Brucella* isolates representing all known species and biovars, and including all type strains, to describe the global population structure of the genus. The collection divided into 101 BruMLSA21 STs (Table S2) with the number of alleles varying from 10 in the case of *caiA* and *fbaA* to 27 in the case of *glk*, a gene included in BruMLSA9, and previously shown to be the most variable in this scheme. Strains of *B. vulpis* and the *B. inopinata*-like isolate BO2 failed to amplify a product at one locus (*mviM*) and were not assigned a BruMLSA21 ST although they have a full BruMLSA9 profile and ST. In addition the application of BruMLSA9 to a larger isolate collection than previously increased the number of BruMLSA9 STs from 27 to 60 when applied to the strain collection described here (Table S2).

### Overarching population structure

**Figure 1:**
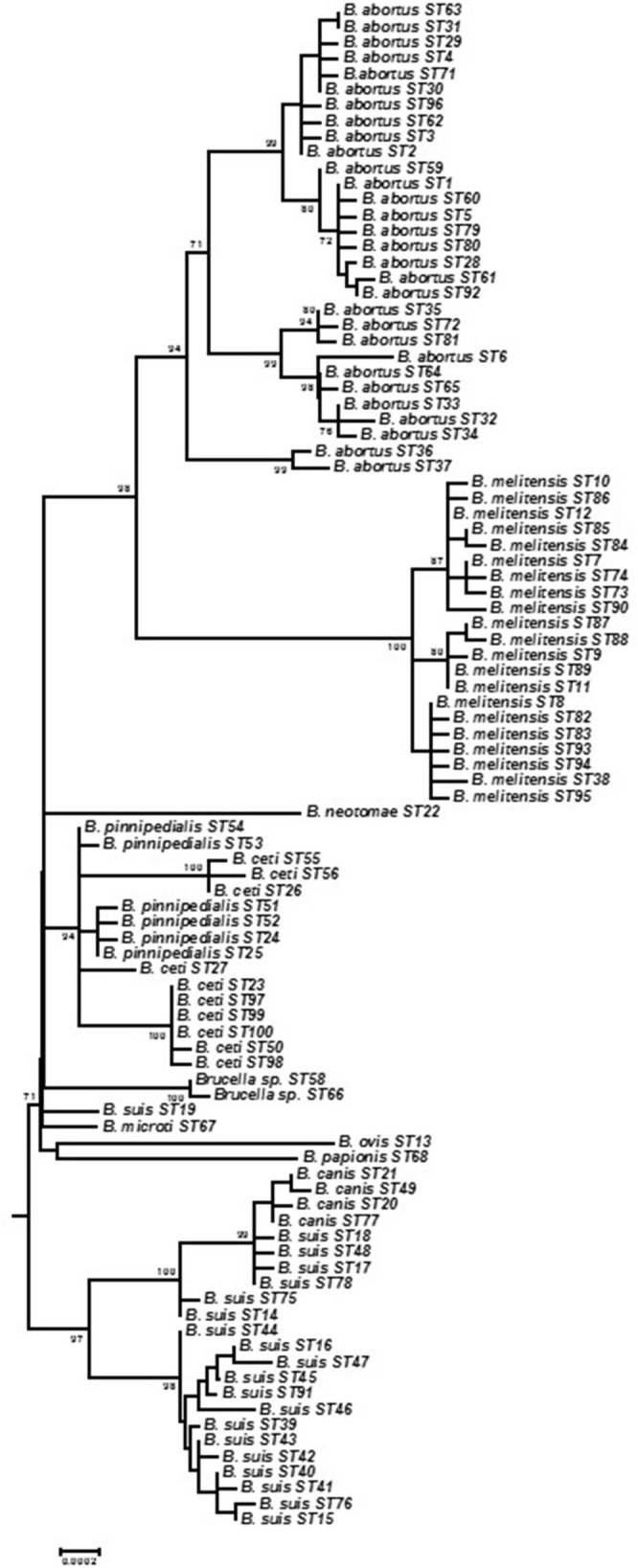
**Expanded subtree of phylogenetic relationships between BruMLSA21 STs based on concatenated sequence data and excluding atypical *Brucella* (see Figure S1) to facilitate better separation of branches**. Bar = nucleotide substitutions per site.

Individual allele sequences for each individual ST were concatenated to form a 10,257 bp length sequence for analysis with 493 (4.81%) sites being polymorphic excluding indels. With 219 polymorphic sites (2.14%) diversity is much reduced if only the core *Brucella* group are considered (i.e., without BruMLSA21 STs 57, 69, 70, and 101 representing *B. inopinata* and unclassified isolates from Australian rodents). Phylogenetic analysis based on all 101 STs is shown in Figure S1 and illustrates the overall structure of the genus with *B. inopinata* and isolates from Australian rodents comprising early branching groups in the genus as currently described. Studies of a number of other groups including isolates from frogs (Eisenberg et al., 2012; Whatmore et al., 2015; Scholz et al., accepted) and foxes (*B. vulpis*) (Scholz et al., 2016) for which BruMLSA9, or partial BruMLSA21, data are available confirm that these isolates also belong to the genetically atypical *Brucella* but that they are clearly much more closely affiliated to the genus *Brucella* than to the nearest phylogenetic neighbor *Ochrobactrum*. Figure 1 shows the core *Brucella* only as an expanded subtree to facilitate resolution. BruMLSA21 confirms the separation into clear clades comprising *B. abortus*/*B. melitensis, B. suis/canis, B. ceti/B. pinnipedialis, B. ovis/B. papionis*, and *B. neotomae*. These clades appear to have radiated virtually simultaneously from a common ancestor into host specific lineages. This was previously also seen with BruMLSA9 analysis and it was initially hoped that the additional discrimination offered by BruMLSA21 would help resolve this. However, it is now apparent that even whole genome sequencing phylogenies suggest the same explosive radiation has occurred although the reasons for this remain unclear (Audic et al., 2011; Wattam et al., 2014). Two additional short branches represent *B. suis* biovar 5 (BruMLSA21 ST19) and *B. microti*—the greater resolution offered by whole genome sequencing has suggested that the former is a very early branching group of the *B. suis*/*B. canis* lineage while the latter is just basal to the classical *Brucella* lineage (Audic et al., 2011; Wattam et al., 2014).

One additional lineage corresponds to BruMLSA21 STs 58 and 66—these represent historical isolates obtained by APHA in the 1960s reportedly associated with human infections in Thailand and originally characterized as atypical *B. suis*. These isolates might, with additional analysis, merit description as a novel species and illustrate the value of MLSA in placing novel isolates in the context of the complete extant understanding of the population structure of the genus. Such data adds to the evidence base facilitating description of novel species as has been the case for *B. papionis, B. inopinata, B. microti*, and *B. vulpis* (Scholz et al., 2008, 2010, 2016; Whatmore et al., 2014) and for other emerging atypical *Brucella* awaiting formal taxonomic description (Tiller et al., 2010a; Eisenberg et al., 2012; Whatmore et al., 2015).

**Figure 2:**
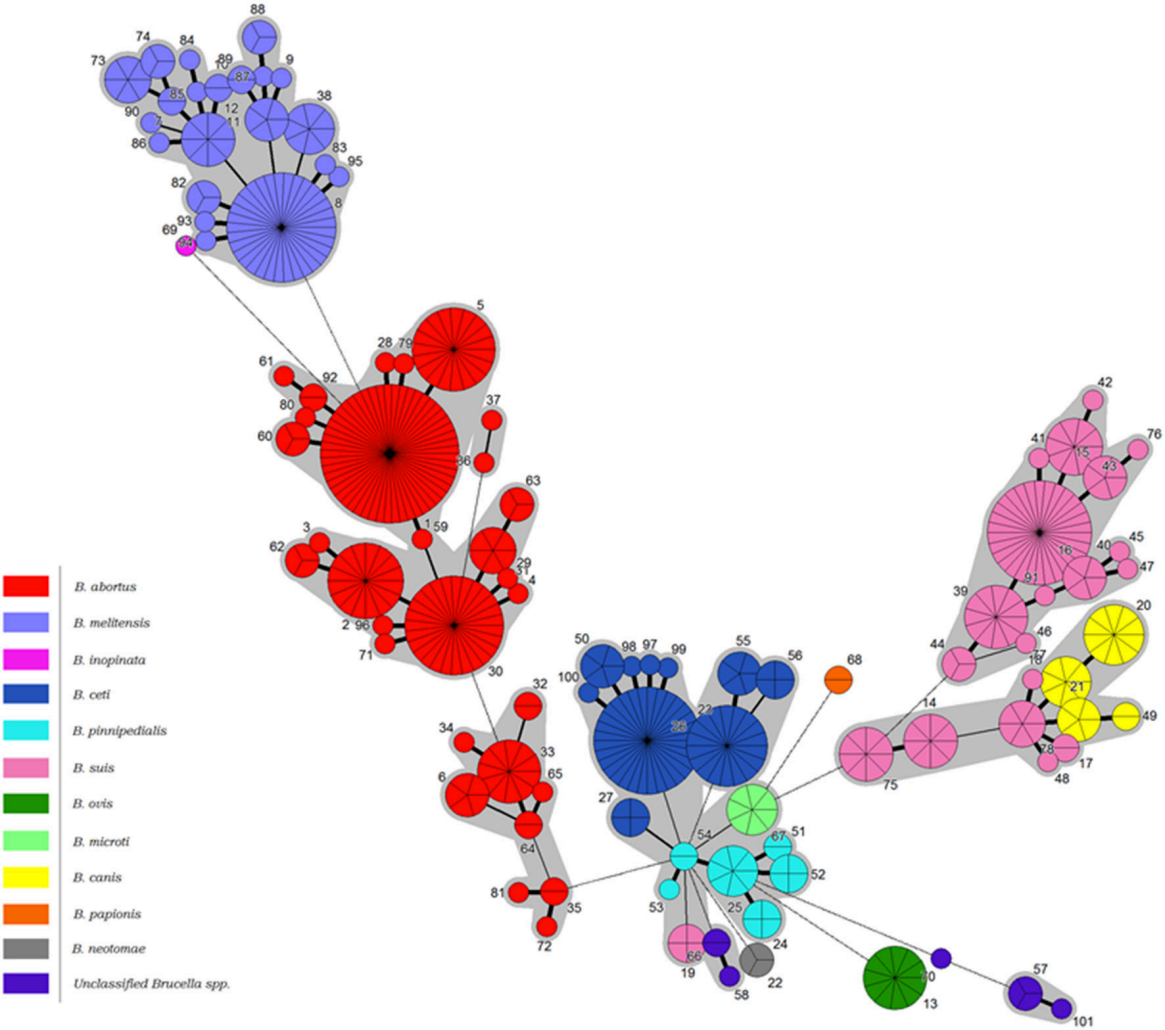
**Minimum spanning tree of BruMLSA21 profiles described in this study**. Each circle denotes a particular ST type with the size of the circle illustrating the number of isolates of that particular type. The coloring inside the circles indicates the *Brucella* species. Thick solid lines joining two types denote types differing at a single locus, thinner solid lines types differing at two or three loci, and the thinnest solid lines types differing at four or five loci. Dashed lines indicate types differing at >5 loci. The gray halos surrounding groupings represent clusters defined in Bionumerics created if neighbors differed in no more than 5 of 21 loci.

Construction of a minimum spanning tree treating data categorically and defining clusters on the basis of neighbors differing in no more than five of the 21 loci revealed a similar relationship (Figure 2). Under these analysis conditions *B. melitensis* constituted a single complex, the more diverse *B. abortus* three complexes corresponding to Clades A, B, and C described below by intraspecies phylogenetic analysis, and *B. suis* biovars 1–4/*B. canis* two complexes, one corresponding to biovar 2 and the other biovars 1, 3, and 4 and *B. canis*. *B. papionis, B. ovis*, and *B. neotomae* all constitute separate groups while the remaining major central complex contains *B. ceti*. *B. pinnipedialis, B. microti*, and *B. suis* biovar 5 isolates.

### Intraspecies relationships within *B. abortus:* phylogeography and biovar

**Figure 3:**
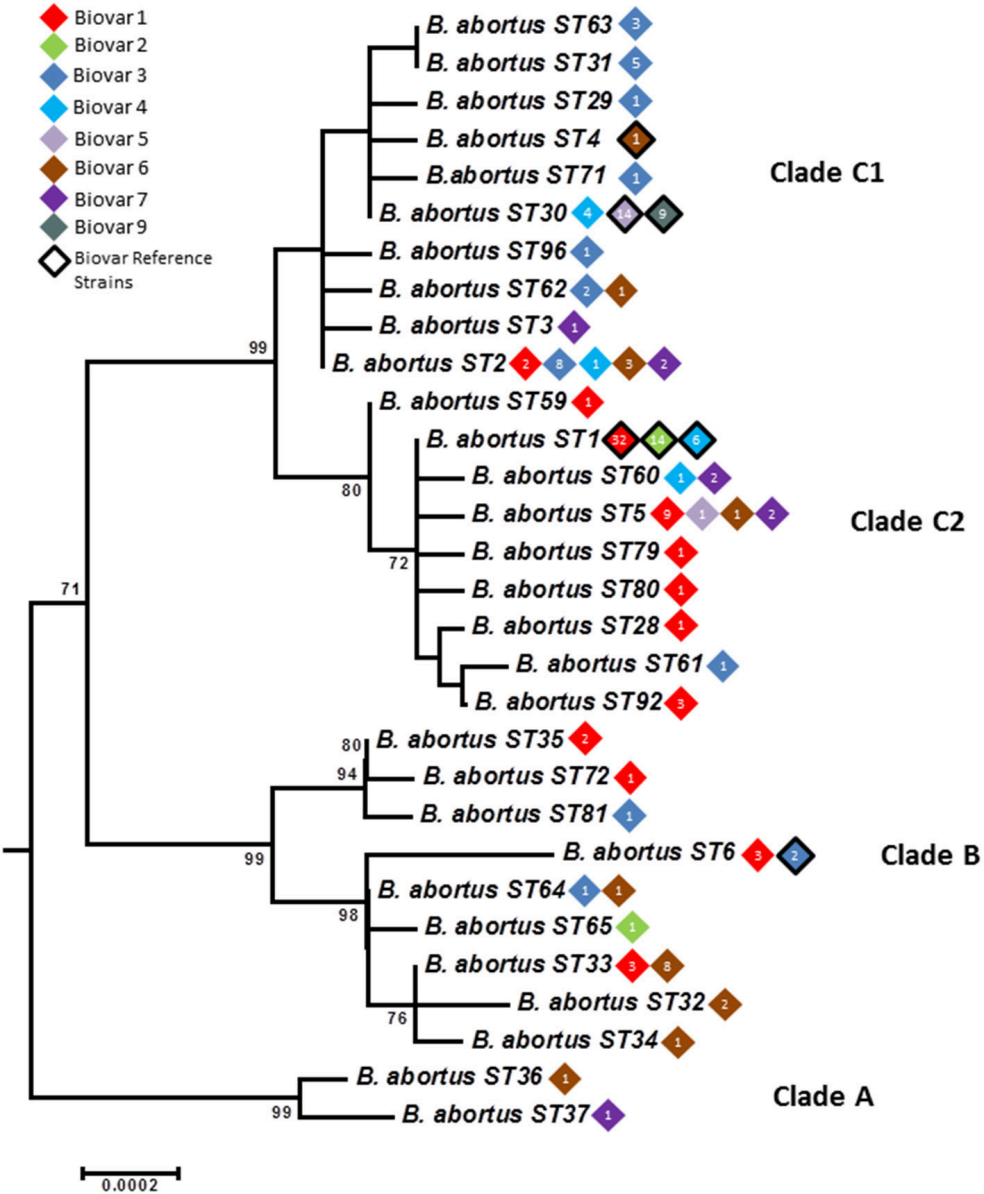
**Intraspecific relationships within *B. abortus***. Expanded subtree (from Figure S1) of the *B. abortus* lineage based on concatenated sequence data highlighting the relationship between ST and biovar. The number inside the rhombus represents the number of isolates. Bar = nucleotide substitutions per site.

**Figure 4:**
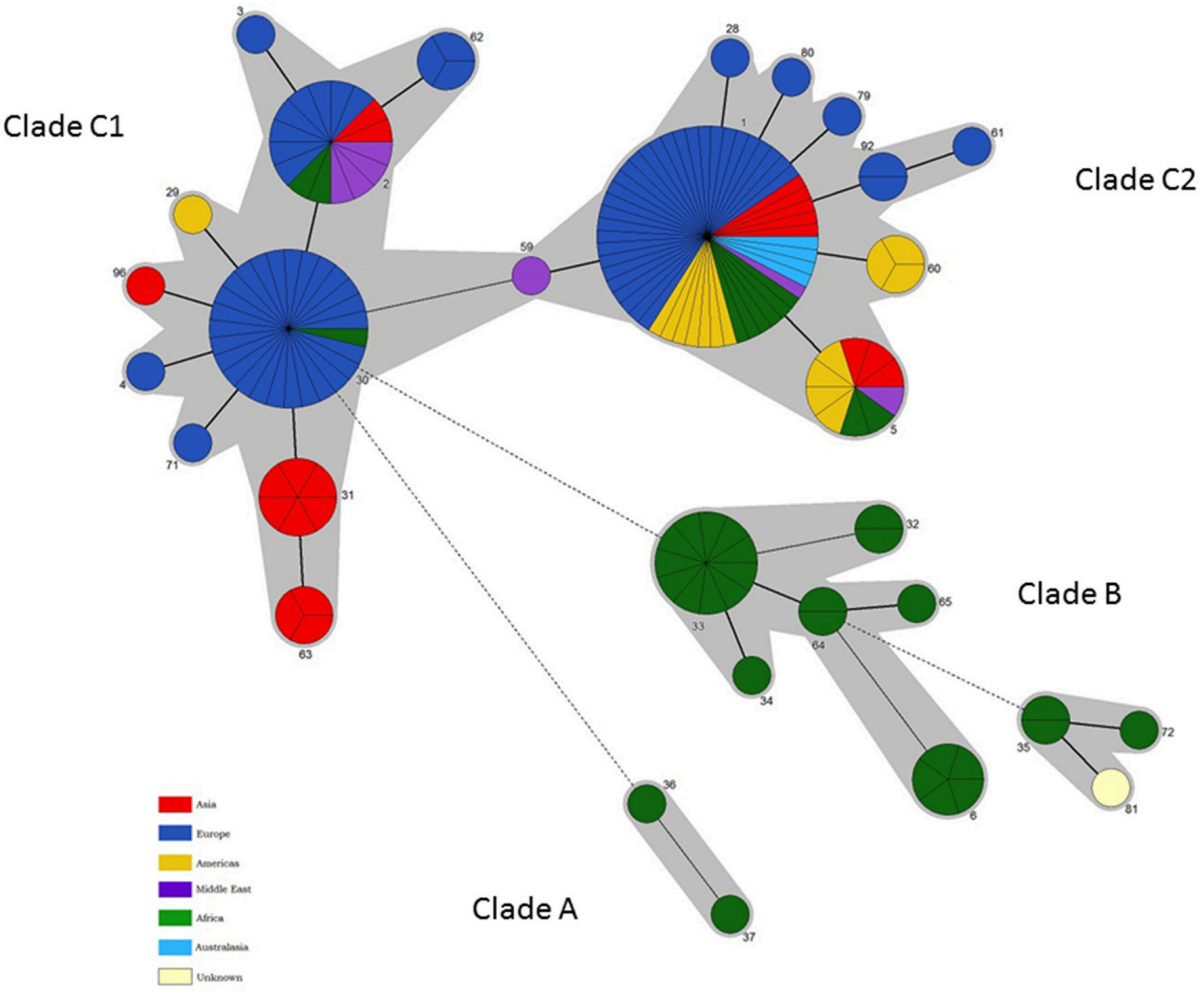
**Intraspecific relationships within *B. abortus*.** Minimum spanning tree showing the relationships between STs with the geographical origin (continent) of isolates highlighted. Each ST is represented by a circle the size of which reflects the number of isolates of that ST in the population analyzed here. Thick solid lines joining two types denote types differing at a single locus, thinner solid lines types differing at two or three loci, and dashed lines types differing at four or more loci. The gray halos surrounding groupings represent clusters defined in Bionumerics created if neighbors differed in no more than 3 of 21 loci.

Only six *B. abortus* STs were described initially by BruMLSA9—application of BruMLSA21 to a wider strain collection significantly increased known diversity of *B. abortus* with 30 STs identified. Isolates comprise three major clades A, B, and C (Figures 3, 4)—the two early branching clades A and B comprise isolates entirely originating from widely across Africa (Senegal, Nigeria, Zimbabwe, Sudan, Mozambique, Kenya, Chad, and Uganda) with clade A representing a previously undescribed clade that substantially increases known diversity of *B. abortus*. These clades consist predominantly isolates of biovars 1, 3, and 6 including the biovar 3 reference strain Tulya.

In contrast to the geographical restriction of clades A and B, isolates of clade C have a global distribution with in particular ST1 and ST2 appearing to be widely distributed across many continents. As shown in Figure 3 this clade splits into two subclades—in clade C1 the most common genotype to biovar association is with biovar 3. However one ST, ST30 is associated exclusively with isolates of biovars 4, 5, and 9 and includes virtually all isolates of the latter two biovars and their reference strains. The vast majority of these isolates originate from the UK (prior to brucellosis eradication) with most of the few remaining isolates from other parts of Western Europe. Consistent with this biovar 5 was colloquially known as “*British melitensis*” (Corbel and Banai, 2005) and is differentiated from biovar 9, colloquially known as “*H*_2_*S producing melitensis*” (Corbel and Banai, 2005), only by H_2_S production. The remaining isolates of ST30 represent biovar 4 which is also seen in genetically distinct lineages and differs from biovar 5 and 9 only it its inability to grow in the presence of thionin. While the overall picture within Clade C is of global dispersal there is evidence that individual genotypes may reflect types with restricted geographical range that may be endemic in certain regions—notably STs 63 and 31 were exclusively associated with East Asia (India, Sri Lanka, and Thailand). Much more detailed local analysis, enabled by the work described here, will help better understand the genotypes prevalent at local levels.

Clade C2 appears more strongly associated with biovar 1 isolates. The particularly widely dispersed genotype, ST1, consists of isolates of biovar 1, 2, and 4 including the reference strains for all these biovars. Biovars 1 and 2 differ only in fuchsin susceptibility while biovar 4 isolates show a different pattern of agglutination with monospecific sera. *B. abortus* biovars 1, 2, and 4 have been shown historically to be closely genetically related (e.g., Gargani and López-Merino, 2006) and, based on these findings it is still not possible to identify distinct genetic lineages corresponding to these biovars.

In summary these data, which reflect the first extensive genetic characterisation of *Brucella* from diverse African origins, suggest that *B. abortus* originated in Africa and, the fact that isolates of the two earliest emerging branches (clades A and B) are still confined there to date, may suggest no or limited global spread of these lineages. In contrast the later emerging clade C appears to have been spread globally to all six populated continents. Indeed it is interesting to note that sporadic members of this clade have been isolated from Africa which may suggest these newer lineages were introduced to Africa by cattle importation from Europe such that both the original endemic lineages (Clades A and B) and, probably to a much lesser extent, introduced lineages (Clades C1 and C2) may now circulate in Africa.

While some relationship between genotype and biovar is apparent in *B. abortus* the picture is far from one of complete congruence. For example isolates of biovar 1 are widely distributed in clades B and C2 and, to a lesser extent in C1. Most isolates in the major early diverging African clade correspond to biovars 1, 3, and 6 (with the latter two biovars separable only by the requirement of biovar 3 for additional CO_2_ and use of thionin at 1/25,000 dilution) although, of the reference strains, only that of biovar 3 is found in this clade. Notably clade C1 is also strongly associated with biovar 3—these data appear consistent with reports in the literature of two biovar 3 clades, 3a from Africa (our Clade B) and 3b (our Clade C1) from Europe (Ocampo-Sosa et al., 2005; Ica et al., 2008; Bertu et al., 2015). Biovar 3b isolates have been reported from Spain and Turkey consistent with the sources of isolates falling within clade C1 (Table S2). In contrast, biovars 5 and 9 were almost entirely confined to a single ST within clade C1 suggesting these biovars are congruent with a distinct genetic lineage although clearly genetic separation between these two biovars was not possible using this MLSA scheme. The biovar 6 reference strain is also located in clade C1 albeit with a unique genotype, ST4, consistent with the description of a PCR assay specific for *B. abortus* biovars 3b, 5, 6, and 9 although this notably was only applied to reference strains of the latter three biovars (Ocampo-Sosa et al., 2005). Readers should note that the original biovar 7 reference strain (63/75) has been shown to represent a mixed culture and biovar 7 was thus deleted from *Brucella* taxonomy (Corbel, 1988; Garin-Bastuji et al., 2014). However genetically diverse isolates that possess the phenotypic profile reported for biovar 7 have been included here and reported in other studies (Garin-Bastuji et al., 2014; Kim et al., 2016). The genetic diversity of strains with the biovar 7 phenotype questions any move to reintroduce biovar 7 as previously defined. Of the remaining biovars, biovar 2 is almost exclusively associated with ST1 within clade C2 that also includes the majority of biovar 4 strains and a large number of biovar 1 strains. The reference strains for all three biovars 1, 2, and 4 are members of ST1 highlighting the fact that it not possible to identify defined and unique genetic lineages that correspond to these biovars.

Finally both *B. abortus* vaccine strains S19 and RB51 were found to be members of ST5 consistent with the identification of North American field isolates in this ST (the parental strains of the vaccines were North American isolates). Given the sharing of this ST between field and vaccine strains ST5 strains were examined by molecular assays to determine status as vaccine or field strains (Sangari and Agüero, 1994; Vemulapalli et al., 1999; Gopaul et al., 2010). These analyses identified that a substantial proportion of isolates in this ST appear to represent S19 vaccine re-isolated from the field (Table S2).

### Intraspecies relationships within *B. melitensis*: phylogeography and biovar

**Figure 5:**
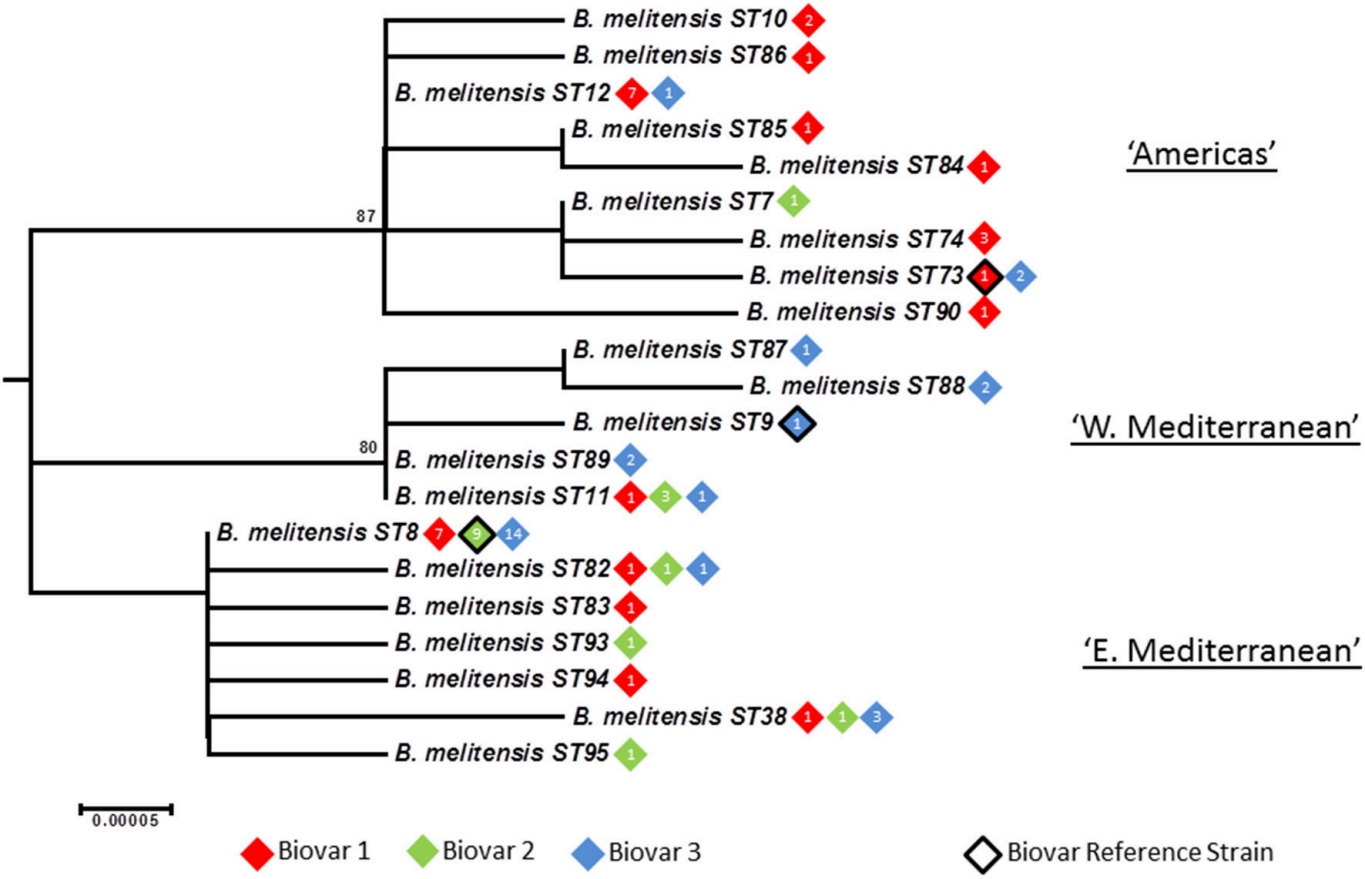
**Intraspecific relationships within *B. melitensis***. Expanded subtree (from Figure S1) of the *B. melitensis* lineage based on concatenated sequence data highlighting the relationship between ST and biovar. The number inside the rhombus represents the number of isolates. Bar = nucleotide substitutions per site.

**Figure 6:**
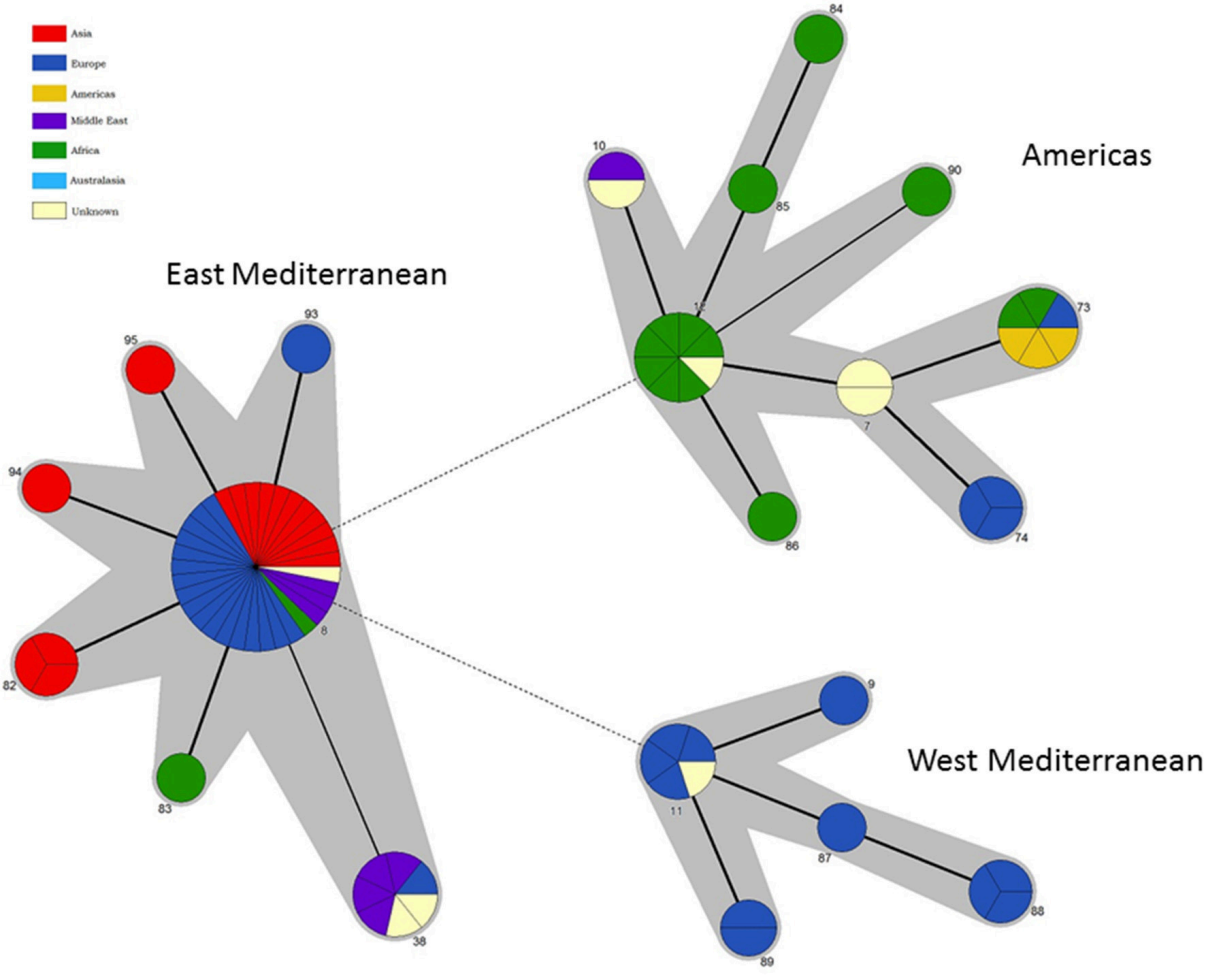
**Intraspecific relationships within *B. melitensis***. Minimum spanning tree showing the relationships between STs with the geographical origin (continent) of isolates highlighted. Each ST is represented by a circle the size of which reflects the number of isolates of that ST in the population analyzed here. Thick solid lines joining two types denote types differing at a single locus, thinner solid lines types differing at two loci, and dashed lines types differing at three or more loci. The gray halos surrounding groupings represent clusters defined in Bionumerics created if neighbors differed in no more than 2 of 21 loci.

Application of BruMLSA21 to a wider *B. melitensis* strain collection identified 21 STs in contrast to the 6 STs previously described by BruMLSA9. Isolates cluster into three distinct lineages (Figures 5, 6) that correspond to the “Americas,” “West Mediterranean,” and “East Mediterranean” lineages described previously on the basis of MLVA data (Al Dahouk et al., 2007). Only a small number of isolates from the Americas were examined here and while isolates from USA and Argentina are included in the Americas clade more than 60% of the isolates of this clade are of African origin. African isolates included in this clade originate widely across the continent (Ethiopia, Somalia, Nigeria, Tanzania, Sudan, Zimbabwe) and, as few African isolates are seen in the “West Mediterranean” and “East Mediterranean” lineages, it appears that this lineage may be endemic in Africa.

In agreement with the previous designation isolates falling in the “West Mediterranean” lineage were exclusively reported from Italy, France, the former Yugoslavia and Germany. In contrast the “East Mediterranean” lineage, while including isolates from Greece, Turkey, Cyprus, and the Balkan States, also extends much more widely into the Middle East and Asia (Thailand, India, Pakistan, Mongolia, and Afghanistan). Although numbers are small some BruMLSA21 STs appear geographically restricted—for example ST82 associated with Thailand (and in agreement with the identification by a recent whole genome sequence analysis of a separate lineage within the “East Mediterranean” cluster including the ST82 defining strain and strains from Malaysia and the Philippines—Tan et al., 2015) and ST38 to the Middle East. However ST8, by far the most frequently seen *B. melitensis* ST, appears widely distributed across Europe and Asia from Portugal to Afghanistan and Mongolia.

While each of the three lineages contains one of the *B. melitensis* biovar reference strains (“West Mediterranean” Ether biovar 3; “East Mediterranean” 63/9 biovar 2; “Americas” 16M biovar 1) there is no clear relationship between genotype and biovar. While the majority of isolates in the “Americas” lineage belong to biovar 1 (81%) and in the “West Mediterranean” lineage belong to biovar 3 (64%), isolates of both other biovars were apparent in both of these lineages. In addition virtually equivalent numbers of all three biovars are seen in both the “East Mediterranean” lineage overall and the most common and widely dispersed BruMLSA21 ST8 specifically. Early MLVA studies highlighted a lack of congruence between genotype and biovar (Whatmore et al., 2006) and a growing body later MLVA studies (e.g., Al Dahouk et al., 2007; Jiang et al., 2011, 2013), taken together with data presented here using markers more appropriate for determining phylogeny, suggest that the biovar concept, which is the case of *B*. *melitensis* is reliant solely on serological reaction, is of extremely limited epidemiological value for *B. melitensis*.

### Intraspecies relationships of *B. suis/B. canis* lineage: phylogeography and biovar

**Figure 7:**
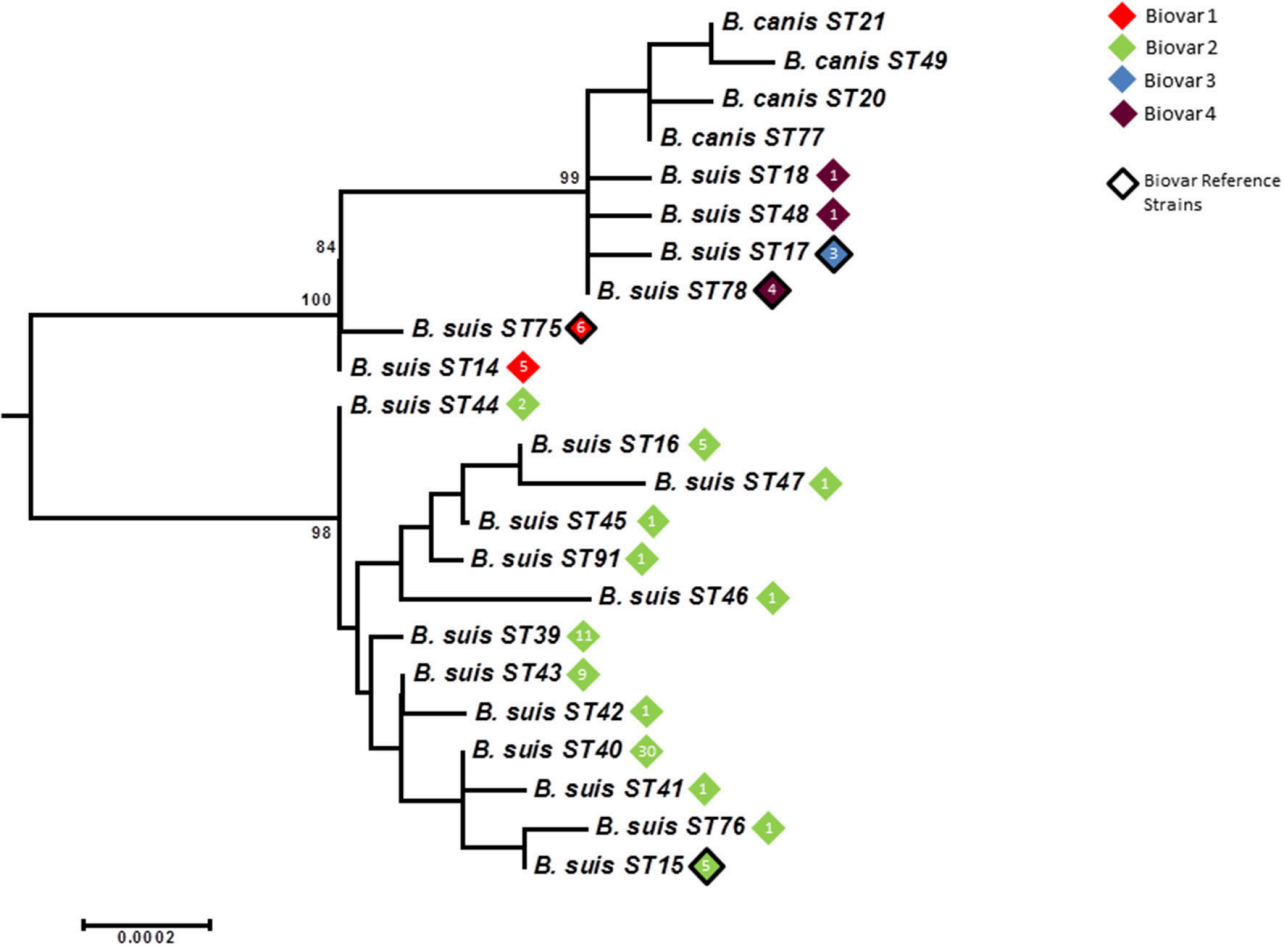
**Intraspecific relationships within the *B. suis/B. canis* lineage**. Expanded subtree (from Figure S1) of the *B. suis/B. canis* lineage based on concatenated sequence data highlighting the relationship between ST and biovar. The number inside the rhombus represents the number of isolates. Bar = nucleotide substitutions per site.

As with other species application of BruMLSA21 to a wider *B. suis* strain collection significantly increased the number of STs identified in BruMLSA9 from 6 to 20. In contrast to *B. abortus* and *B. melitensis* there is a strong congruency between genotype and biovar. Excluding *B. suis* biovar 5 there are two genetically divergent major clades (Figure 7), one comprising to biovar 2 isolates and the second comprising an early branching group corresponding to biovar 1 isolates, biovar 3 and 4 isolates and *B. canis* isolates forming a terminal group on this branch. Thus only biovar 3 and 4 isolates do not represent clearly separated clusters although, in contrast to BruMLSA9 where they share a genotype, the small number of biovar 3 isolates included do correspond to a single unique BruMLSA21 ST (ST17).

**Figure 8:**
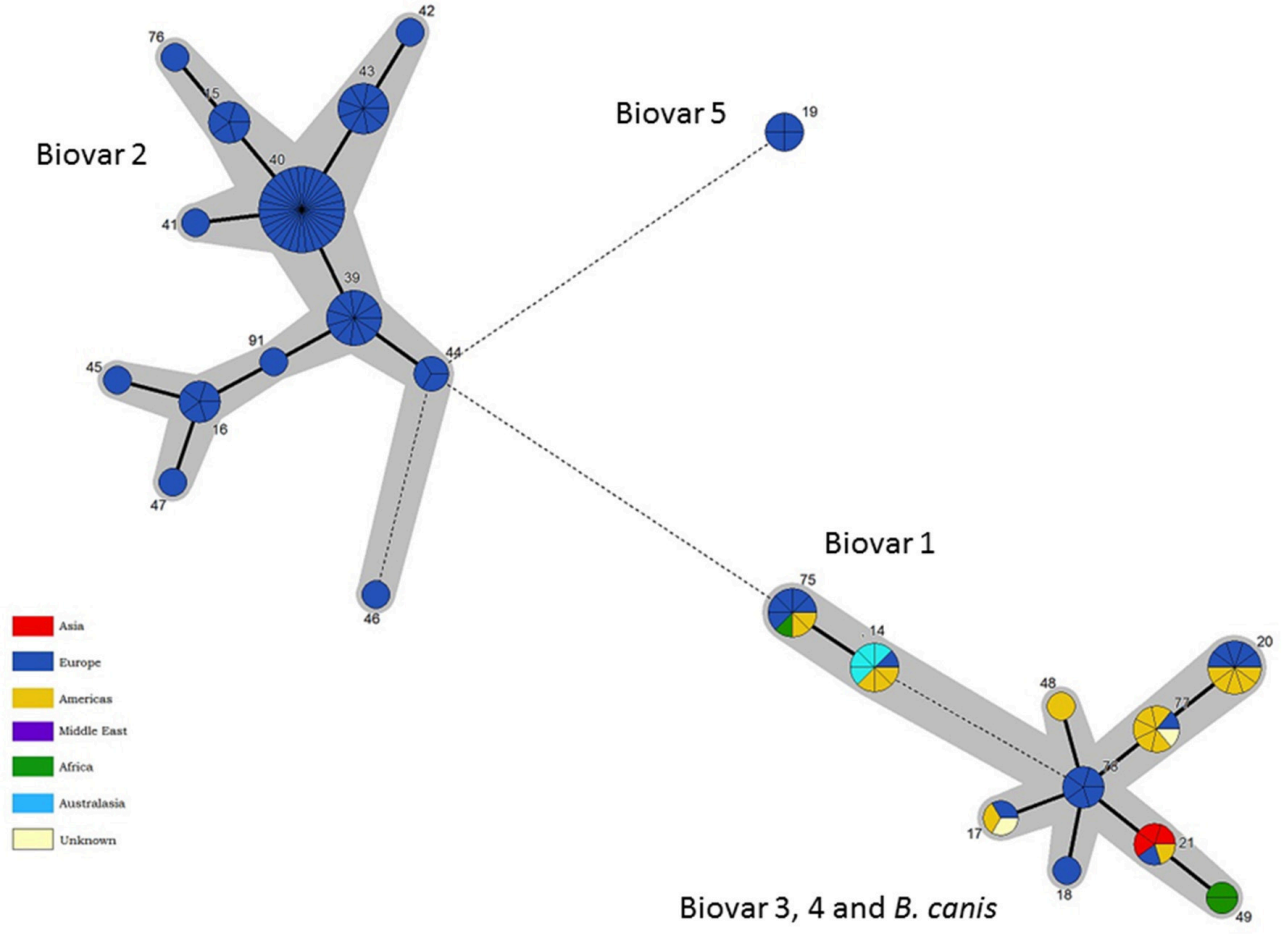
**Intraspecific relationships within the *B. suis/B. canis* lineage**. Minimum spanning tree showing the relationships between STs with the geographical origin (continent) of isolates highlighted. Each ST is represented by a circle the size of which reflects the number of isolates of that ST in the population analyzed here. Thick solid lines joining two types denote types differing at a single locus, thinner solid lines types differing at two to four loci, and dashed lines types differing at >4 loci. The gray halos surrounding groupings represent clusters defined in Bionumerics created if neighbors differed in no more than 4 of 21 loci.

Only biovar 2 has sufficient isolates to ascertain possible phylogeographic associations (Figure 8). Here the vast majority of STs are associated with Central and Northern European isolates where it is likely that movement of strains in wildlife reservoirs (hares and wild boar) has dispersed genotypes widely. However two genotypes ST39 and ST44 are confined to the Iberian peninsula suggesting they correspond to the Iberian clone of *B. suis* biovar 2 described previously on the basis of PCR-RFLP, MLVA and subsequent whole genome mapping (Ferreira et al., 2016).

### Intraspecies relationships of *B. pinnipedialis* and *B. ceti*

**Figure 9:**
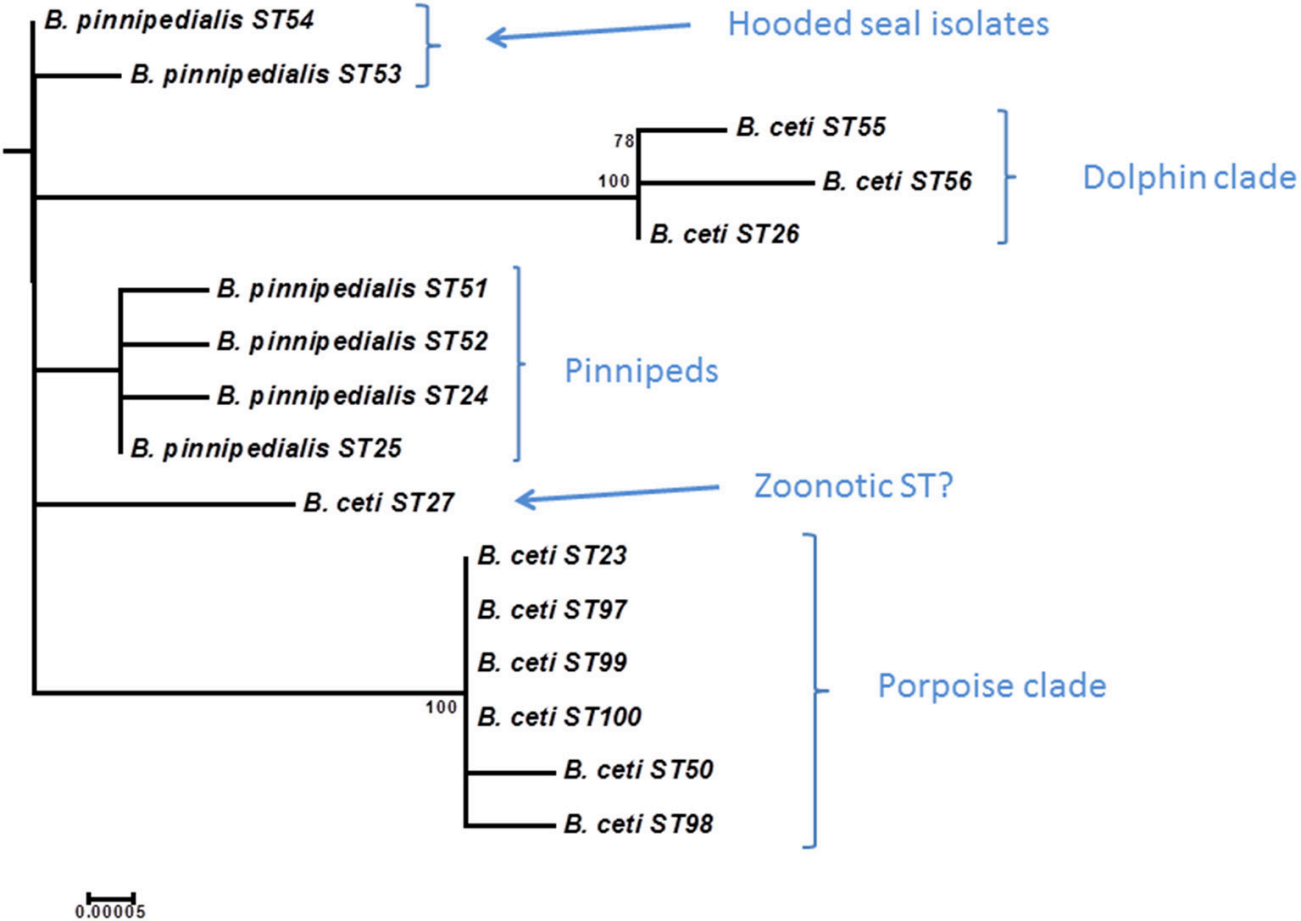
**Expanded subtree (from Figure S1) of the marine mammal *Brucella* lineage based on concatenated sequence data**. Bar = nucleotide substitutions per site.

Grouping of marine mammal *Brucella* remains largely consistent with that described by BruMLSA9 with an increase in STs from 5 to 16 (Figure 9 and Table S2). As suggested previously phylogeny appears inconsistent with taxonomy in this group and *B. ceti* appears paraphyletic with two distinct clusters (ST26 complex and ST23 complex) that appear to have preferred hosts of dolphins or porpoises, respectively (Groussaud et al., 2007; Dawson et al., 2008). The increased resolution of BruMLSA21 allows further subdivision of isolates of BruMLSA9 ST24 and ST25 associated with various species of seals for example identifying two BruMLSA21 STs (ST53 and ST54) that appear to be specific for hooded seals in this dataset, a finding consistent with the previous description of a hooded seal specific clade on the basis of MLVA (Groussaud et al., 2007; Maquart et al., 2009). The remaining ST, ST27 has previously been associated with human zoonotic infection (Whatmore et al., 2008) and remains distinct from the main marine mammal *Brucella* clusters. These data again highlight that phylogenetic relationships suggest that a taxonomic rearrangement of *Brucella* from marine mammals might be appropriate (Whatmore, 2009).

### Remaining species

No diversity was identified in *B. microti, B. neotomae, B. papionis*, and *B. ovis*. In large part this may reflect the very limited sampling of these rarely or recently isolated species although *B. ovis* has been more extensively sampled with isolates from across the globe included here. These data, and complementary MLVA data, which also reveals relatively limited diversity within *B. ovis* (Whatmore et al., 2006), suggest that *B. ovis*, while relatively well-separated from other classical species, may represent an extremely genetically restricted group.

Only a single isolate of *B. inopinata* has been formally described although the increasing isolation of diverse genetically atypical *Brucella* from various hosts might point toward consideration of whether the description of *B. inopinata* should be expanded to include all these early-branching *Brucella* isolates.

## Conclusions

Although we have previously described the application of BruMLSA21 to address specific issues, for example in the context of addressing the status of *B. abortus* biovar 7 (Garin-Bastuji et al., 2014), full technical details of the scheme, allele calls, ST allelic profiles, and associated metadata have not previously been available to facilitate comparative analysis by others.

To bring *Brucella* into alignment with most other bacterial pathogens the existing BruMLSA9 data (Whatmore et al., 2007) and BruMLSA21 data described here, as well as a large volume of additional unpublished BruMLSA9 data has now been made available via the principle MLST/MLSA database as a resource for the scientific community. The full data set, sampling c. over 740 isolates for BruMLSA9 and more than 500 isolates for BruMLSA21, provide the most comprehensive assessment of the population structure of the entire genus to date. They provide a framework for understanding the relationship between extant strains, for the placement of newly emerging or discovered strains in the context of extant knowledge and a platform for designing and or assessing the performance of molecular diagnostic assays designed to characterize strains at various taxonomic levels.

The study described here adds to understanding of the phylogeographical relationships within the major *Brucella* species for the first time clearly describing the apparent origin of *B. abortus* in Africa and the global spread of later emerging lineages. Completely novel lineages, such as the early emerging African *B. abortus* lineage, have been revealed which may be significant in understanding the emergence of *B. abortus* as a host specific pathogen. Equally there appear to be clones endemic only locally, such as the *B. abortus* ST63/ST31 Asian clone or the Middle Eastern *B. melitensis* ST38, although much of the world, particularly where brucellosis is most problematic, remains grossly under sampled with insufficient data to draw robust conclusions. It is hoped the publication of these data encourage the application of MLSA (either *per-se* or using data extracted from whole genome sequences) more widely to expand the depth of the database described here and further understanding of the global *Brucella* situation.

The data also add significantly to understanding the relationship between biovar and genotype—it is clear that there is there is diminishing congruence in this relationship from *B. suis* > *B. abortus* > *B. melitensis*. This is not entirely unexpected given a system based on phenotypic or serological markers that may be impacted by factors such as variable expression and the acknowledged subjectivity of the approach combined with the very subtle differences in phenotype of some of the biovars described. Given advances in molecular approaches it would seem timely to consider whether such approaches offer a more meaningful replacement for biotyping. Such assays could be based on genomic rearrangements such as the multiplex Bruceladder (López-Goñi et al., 2008) or SNPs as per existing speciation assays (Scott et al., 2007; Foster et al., 2008; Gopaul et al., 2008) which have the advantage that they can be applied at a number of taxonomic levels (Keim et al., 2004). The crucial point is that they must be designed on the back of a robust population genetic understanding provided by data such as that presented here and iteratively re-examined as knowledge of extant diversity increases. For example it would be very easy to design an assay for *B. abortus* that did not detect the early emerging African lineage if a marker was selected that represented an evolutionary change that occurred beyond this branch.

We welcome submission of new data to the *Brucella* PubMLST website at http://pubmlst.org/brucella/.

## Authors contributions

AW conceived of and designed the study, analyzed the data, and drafted the manuscript. MK, JM, KG, and JE undertook portions of the experimental work and analysis. LP oversaw strain provision and biotyping.

### Conflict of interest statement

The authors declare that the research was conducted in the absence of any commercial or financial relationships that could be construed as a potential conflict of interest. The reviewer VG and handling Editor declared their shared affiliation and the handling Editor states that the process nevertheless met the standards of a fair and objective review.

## Supplementary material

Figure S1 **Phylogenetic reconstruction of relationships between all 101 BruMLSA21 STs including atypical strains**. The tree was constructed with the concatenated sequence data of the 21 loci (>10.2 kb) using the neighbor-joining algorithm with the Jukes-Cantor model in MEGA5.2. Bar = nucleotide substitutions per site.

Table S1 **Technical details of MLSA scheme including details of loci characterized and PCR primer sequences**.

Table S2 **Details of BruMLSA21 data described in this study**. All data have been deposited at http://pubmlst.org/brucella/.
