# Supplementary figures and images for: Extended Multilocus Sequence Analysis to Describe the Global Population Structure of the Genus Brucella: Phylogeography and Relationship to Biovars
Source: Front Microbiol. 2016 Dec 21;7:2049. doi: 10.3389/fmicb.2016.02049 (PMC5174110; doi:10.3389/fmicb.2016.02049)

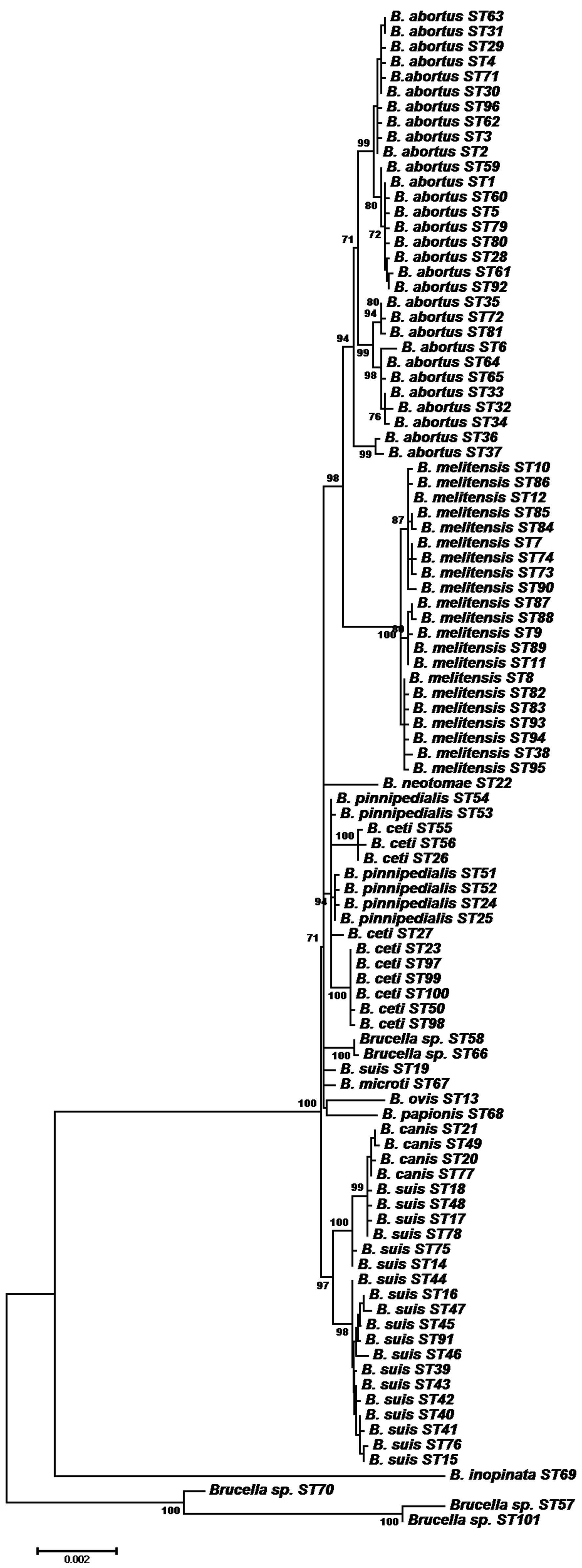

Supplement: Figure S1 — Phylogenetic reconstruction of relationships between all 101 BruMLSA21 STs including atypical strains. The tree was constructed with the concatenated sequence data of the 21 loci (>10.2 kb) using the neighbor-joining algorithm with the Jukes-Cantor model in MEGA5.2. Bar = nucleotide substitutions per site. [file Image1.TIF]
