# Supplementary material for: Extended Multilocus Sequence Analysis to Describe the Global Population Structure of the Genus Brucella: Phylogeography and Relationship to Biovars
Source: Front Microbiol. 2016 Dec 21;7:2049. doi: 10.3389/fmicb.2016.02049 (PMC5174110; doi:10.3389/fmicb.2016.02049)
Supplement: Table S1 — Technical details of MLSA scheme including details of loci characterized and PCR primer sequences. [file Table1.DOC]

**Table S1 - PCR Primers for *Brucella* MLSA-9 and MLSA-21 schemes**

**Locus Putative function Primer sequences Core sequence length Location in *B. abortus* 9-941 sequence**

# MLSA-9 (Whatmore *et al*. BMC Microbiol 7:34)

*gap* glyceraldehyde 3-phosphate dehydrogenase 5’ YGCCAAGCGCGTCATCGT 3’ 589 bp AE017223 1685083-1685671

5’ GCGGYTGGAGAAGCCCCA 3’

*aroA* 3-phosphoshikimate1-carboxyvinyltransferase 5’ GACCATCGACGTGCCGGG 3’ 565 bp AE017223 29974-30538

5’ YCATCAKGCCCATGAATTC 3’

*glk* glucokinase 5’ TATGGAAMAGATCGGCGG 3’ 475 bp^#^ AE017224 988660-989134

5’ GGGCCTTGTCCTCGAAGG 3’

*dnaK* chaperone protein 5’ CGTCTGGTCGAATATCTGG 3’ 470 bp AE017223 2066742-2067211

5’ GCGTTTCAATGCCGAGCGA 3’

*gyrB*  DNA gyrase B subunit 5’ ATGATTTCATCCGATCAGGT 3’ 469 bp AE017223 142378-141910

5’ CTGTGCCGTTGCATTGTC 3’

*trpE* anthranilate synthase 5’ GCGCGCMTGGTATGGCG 3’ 486 bp AE017223 1538194-1537709

5’ CKCSCCGCCATAGGCTTC 3’

*omp25* 25 kDa outer-membrane protein 5’ ATGCGCACTCTTAAGTCTC 3’ 490 bp^#^ AE017223 710041-710530

5’ GCCSAGGATGTTGTCCGT 3’

*cobQ* cobyric acid synthase 5’ GCGGGTTTCAAATGCTTGGA 3’ 422 bp^#^ AE017223 1289341-1288920

5’ GGCGTCAATCATGCCAGC 3’

int*-hyp* upstream and extreme 5’ of hypothetical protein 5’ CAACTACTCTGTTGACCCGA 3’ 430 bp^#^ AE017223 1372708-1372279

5’ GCAGCATCATAGCGACGGA 3’

# Additional loci for MLSA-21

*prpE* propionate-CoA ligase 5’ ggtgctgttcacgctggaa 3’ 468 bp AE017223 1687838-1688305

5’ aggttttcgcaggcggcgaa 3’

*caiA* acyl-CoA dehydrogenase 5’ tgtgttcggcaagcctttg 3’ 449 bp AE017224 633492-633940

5’ ggtcaaaagacgtgccaca 3’

*csdB* cysteine desulfhydrase 5’ CGTCACTTCCTGGATCATTTC 3’ 487 bp AE017223 930109-929623

5’ GCCACCGACGCTTATGAGAA 3’

*soxA* sarcosine oxidase alpha subunit 5’ cctcgtaaagcgccttcc 3’ 486 bp AE017223 245488-245003

5’ tgttcgatgcctccacattgg 3’

*leuA* 2-isopropylmalate synthase 5’ tcaaccggatgaaggaagtc 3’ 482 bp AE017223 1534688-1534207

5’ ccctcgatagtcttggtgaca 3’

*mviM*  glucose-fructose oxidoreductase precursor 5’ ATCGCCCGTTCGGTGAC 3’ 447 bp^#^ AE017223 1990600-1990154

5’ TGTTCGCCGTCCTTGTCC 3’

*fumC* fumarate hydratase C 5’ cgaccatgtcaatatgagcc 3’ 452 bp AE017224 180504-180055

5’ gatatcgttggcgatcttgaa 3’

*fbaA* fructose-bisphosphate aldolase 5’ cgtgaaataacctgatctcac 3’ 458 bp AE017224 360206-360663

5’ catgccggtttcaagcgaac 3’

*ddlA* D-alanine-D-alanine ligase A 5’ tttcagtgcgctcgaacag 3’ 553 bp AE017223 1251848-1251296

5’ gttcttcaatgatgagattaaa 3’

*putA* proline dehydrogenase 5’ gtgggcgtgcagcctttcg 3’ 527 bp AE017224 512957-513483

5’ cctgtgtgagtacgagcgg 3’

*mutL* DNA mismatch repair protein 5’ ACATCCAAGCTGACCGAC 3’ 549 bp AE017224 208009-207461

5’ TCCCGTGCGATCACATCCGA 3’

*acnA* aconitate hydratase 5’ GAAGGCCGCATCCCACTG 3’ 490 bp AE017223 99997-99508

5’ GCGGCGAGGCAAGGTAAT 3’

^#^Size variants identified at these loci.
